# Comparison of a curved forceps with a conventional straight forceps for nasogastric tube insertion under videolaryngoscopic guidance: A randomized, crossover manikin study

**DOI:** 10.1097/MD.0000000000007983

**Published:** 2017-09-01

**Authors:** Kenta Furutani, Tatsunori Watanabe, Yoshinori Kamiya, Hiroshi Baba

**Affiliations:** a Department of Anesthesiology, Uonuma Institute of Community Medicine, Niigata University Medical and Dental Hospital, Minami-Uonuma; b Division of Anesthesiology, Niigata University Medical and Dental Sciences, Niigata City, Niigata, Japan.

**Keywords:** Magill forceps, McGRATH MAC, nasogastric tube, SUZY forceps, videolaryngoscope

## Abstract

**Background:**

Nasogastric tube (NGT) insertion is an easy procedure that can be routinely performed under general anesthesia. However, for difficult cases, there are limited insertion techniques available in routine clinical practice, considering the flexibility of NGTs. The SUZY curved forceps are designed for the removal of pharyngolaryngeal foreign bodies under guidance of the McGRATH MAC (McG) videolaryngoscope. Because McG enables clear visualization of the esophageal inlet, we hypothesized that the SUZY forceps can facilitate easier NGT insertion compared with the conventional Magill forceps under McG guidance and designed a randomized, crossover manikin study to test this hypothesis.

**Materials and Methods:**

Ten anesthesiologists participated in this study. Each participant was instructed to insert an NGT using either the SUZY or the Magill forceps under McG guidance. Both types of forceps were used by each participant in a computer-generated random order. The primary outcome measure was the number of “strokes” (1 stroke was defined by a specific sequence of participant actions) required to advance the NGT 30 cm from the starting point. Data are expressed as medians (interquartile ranges [ranges]).

**Results:**

The number of strokes required for NGT insertion was fewer in the SUZY group than in the Magill group {7 [7.0–12.5 (5–14)] vs 16.5 [13.5–20.3 (7–22)]; *P* <.05}. The time required for NGT insertion was also lesser in the SUZY group than in the Magill group {15.4 [13.7–20.0 (7.0–38.3)] seconds vs 30.3 [22.0–42.3 (12.8–47.5) seconds]; *P* <.05}.

**Conclusions:**

The SUZY curved forceps facilitated NGT insertion more effectively than the Magill straight forceps under McG guidance. Our results suggest that NGT insertion using the SUZY forceps under McG guidance is a secure and easy procedure.

## 1 Introduction

The insertion of a nasogastric tube (NGT) is an easy procedure that can be routinely performed under general anesthesia. However, for difficult cases, there are limited insertion techniques available in routine clinical practice, considering the flexibility of NGTs. Failure to insert an NGT can pose a problem in cases where the tube is necessary for surgery or anesthetic management.

Various techniques to facilitate NGT insertion, such as neck flexion with lateral neck pressure,^[1]^ the use of water-filled or frozen NGTs,^[2]^ the use of various types of guidewires,^[1,3,4]^ and inflation of the esophagus with mask ventilation,^[5]^ have been reported. Occasionally, these blind techniques can cause serious complications such as mucosal injury, bleeding, edema, and tracheal misplacement. Therefore, anesthesiologists should avoid blind NGT insertion and perform the procedure under visualization of the larynx and hypopharynx, if possible. Esophageal conduit using an endotracheal tube facilitates NGT insertion.^[6]^ However, the technique is traumatic for the pharyngeal and esophageal mucosa because of the large outer diameter of the conduit. Use of the Magill forceps under guidance of the Macintosh laryngoscope is a popular technique for NGT insertion because it enables direct visualization of the larynx, pharynx, and esophageal inlet. However, it is occasionally difficult to clearly visualize the esophageal inlet with this technique; furthermore, this technique requires a certain amount of skill, particularly when the patient is intubated.

Videolaryngoscopes facilitate easy intubation and also enable clear visualization of both the larynx and esophageal inlet. Some investigators have reported the efficacy of the Pentax-AWS ^[7–9]^ (Hoya, Tokyo, Japan) and Glidescope^[10,11]^ (Verathon, Bothell, WA) for NGT insertion. However, although these videolaryngoscopes enabled visualization of the hypopharynx or the piriform fossa, they could not direct the tip of the NGT to the esophageal inlet, regardless of the attachment of an endotracheal tube guide.^[12]^ Therefore, even these videolaryngoscopes are not considered useful for NGT insertion in difficult cases.

**Figure 1:**
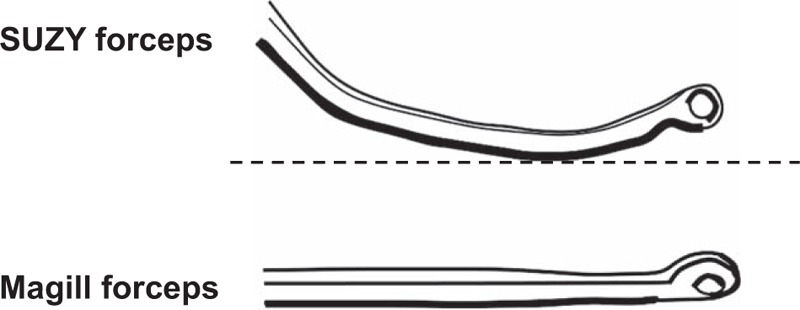
Illustrations of the SUZY curved forceps and conventional Magill straight forceps, The SUZY forceps are curved from the middle to the distal portion and fit the curved blade of the McGRATH MAC videolaryngoscope. The Magill forceps exhibit a straight shape.

The SUZY curved forceps (TDM Corporation, Tokyo, Japan) are designed for the removal of pharyngolaryngeal foreign bodies,^[13,14]^ particularly under guidance of the McGRATH MAC (McG; Aircraft Medical Ltd, Edinburgh, UK) videolaryngoscope. Because the curved form of the SUZY forceps fits the blade of McG (Fig. 1), the tips of the forceps can be clearly visualized on the McG screen. Therefore, we hypothesized that the use of the SUZY forceps under McG guidance may enable easier NGT insertion because of clear visualization of the esophageal inlet and the ease of directing the NGT to the esophagus. However, the safety and efficacy of the SUZY forceps for NGT insertion in humans have not been determined. From this perspective, we performed a preliminary, randomized, crossover manikin study before planning a clinical trial. The aim of the study was to investigate the efficacy of the SUZY forceps in comparison with that of the conventional Magill forceps, which are widely used for NGT insertion or the removal of pharyngeal foreign bodies in conjunction with the Macintosh laryngoscope, for NGT insertion under videolaryngoscopic guidance.

## 2 Materials and methods

### 2.1 Participants

This randomized, crossover manikin study was approved by the Ethics Committee of Niigata University (Niigata, Japan, document No. 2047) and performed in the operating room at Niigata University Medical and Dental hospital in December 2014. Ten anesthesiologists who had performed >300 and >10 tracheal intubation procedures using the Macintosh laryngoscope and McG, respectively, participated in this study. Their clinical experience ranged from 2 to 24 years, and none were familiar with the SUZY forceps. The participants were not informed of the primary or secondary outcome measures of the study. Written informed consent was obtained from all participants.

### 2.2 Preparation

The manikin (Airway Management Trainer, Laerdal, Stavanger, Norway) was placed on a sturdy table. The investigator (KF) inserted the NGT (14 Fr, 122 cm; Argyle stomach tube, Covidien, Dublin, Ireland), which was lubricated with a silicone spray, into the right nostril of the manikin to the level of the piriform sinus under direct visualization using a Macintosh laryngoscope.

Each participant was instructed to insert the NGT using either the SUZY or the Magill forceps (ACOMA medical industry, Tokyo, Japan) under McG guidance. A #3 blade was used. In this crossover study, both types of forceps were used by each participant in a computer-generated random order (via www.randomiser.org). Randomization and allocation were performed and concealed using sealed, prenumbered, opaque envelopes by a single investigator (KF).

### 2.3 NGT insertion and data collection

Before initiating data collection, the participants were instructed to avoid blind manipulation. They were instructed to use both types of forceps in the range of the McG monitor. They were also instructed to insert the NGT as fast as they could, until the tip had advanced 30 cm from the starting point and reached the piriform sinus. They were all asked to confirm whether they could grasp the tip of the NGT using either type of forceps. Thereafter, data collection was initiated. All data were collected by a single investigator (KF). Once all measurements were obtained for 1 type of forceps, measurements for the second type were recorded.

### 2.4 Outcomes

**Figure 2:**
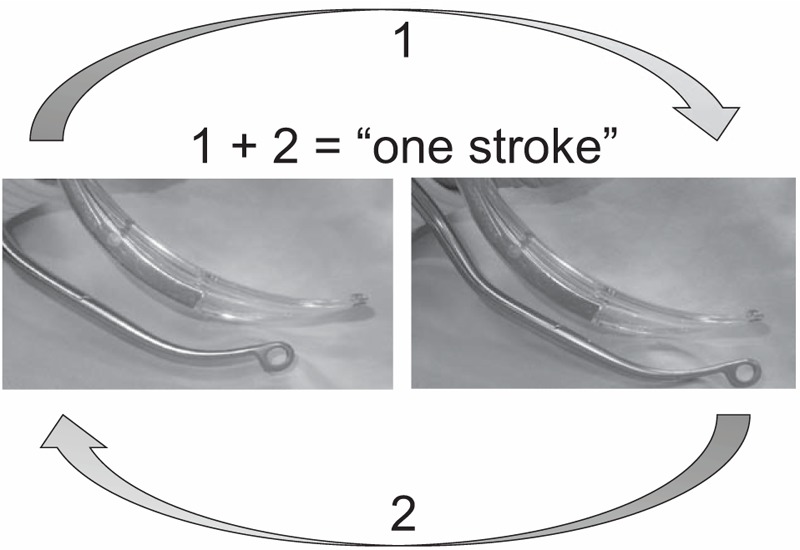
Definition of 1 “stroke” for the insertion of a NGT using forceps in the present study. A stroke was defined as the following sequence of participant actions: grasping the NGT with the forceps, advancing it to the esophageal inlet, and releasing the distal portion of the NGT and grasping the proximal portion again. NGT = nasogastric tube.

We hypothesized that the SUZY forceps would be more effective in NGT insertion under McG guidance compared with the Magill forceps. To evaluate the efficacy of the SUZY forceps, we defined 1 “stroke” as the following sequence of actions performed by the participants: grasping the NGT with the forceps, advancing it to the esophageal inlet, and releasing the distal portion of the NGT and grasping the proximal portion again (Fig. 2). The primary outcome measure was the number of strokes required to advance the NGT 30 cm from the starting point. The secondary outcome measure was the time required to insert the NGT.

### 2.5 Statistical analysis

The sample size was calculated using G-power 3.1 software (Heinrich Heine, University of Düsseldorf, Düsseldorf, Germany). In a pilot study conducted by our colleagues (KF and TW), the mean (±SD) numbers of strokes for NGT insertion in a SUZY group and a Magill group were 12 ± 3.5 and 17 ± 3.5, respectively. A *P*-value of <.05 was considered statistically significant. A power calculation (α = 0.05, β = 0.1) indicated that at least 8 participants were required to detect an improvement of 5 strokes in the SUZY group relative to the number of strokes in the Magill group. Therefore, we recruited 10 participants after considering potential drop-outs due to protocol deviations.

Data were analyzed with GraphPad Prism 6.0 (GraphPad Software, San Diego, CA) software. The number of strokes and the time taken for insertion were analyzed by Wilcoxon signed rank tests. Data are expressed as medians (interquartile ranges [range]).

## 3 Results

**Figure 3:**
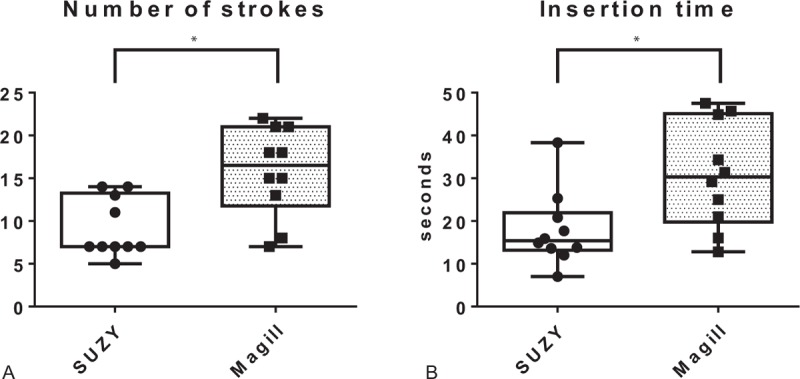
Differences between the SUZY curved forceps and conventional Magill straight forceps for NGT insertion under guidance of the McG videolaryngoscope in this manikin study. The bottom and top of each box represent the first and third quartiles, respectively, and the band inside each box represents the median. The ends of the whiskers represent the maximum and minimum. A: The number of strokes required for NGT insertion is fewer in the SUZY group than in the Magill group. B: The time required for NGT insertion is lesser in the SUZY group than in the Magill group. ^∗^*P* <.05. McG = McGRATH MAC, NGT = nasogastric tube.

No participant was excluded from the study. The mean number of strokes required for NGT insertion was fewer in the SUZY group than in the Magill group {7 [7.0–12.5 (5–14)] vs 16.5 [13.5–20.3 (7–22)]; *P* = .020; Fig. 3A}. The mean time required for NGT insertion was also lesser in the SUZY group than in the Magill group {15.4 [13.7–20.0 (7.0–38.3)] seconds vs 30.3 [22.0–42.3 (12.8–47.5)] seconds; *P* = .014; Fig. 3B}.

## 4 Discussion

In this randomized, crossover manikin study, the SUZY curved forceps decreased the number of strokes and the time required for NGT insertion compared with the Magill forceps under McG guidance.

**Figure 4:**
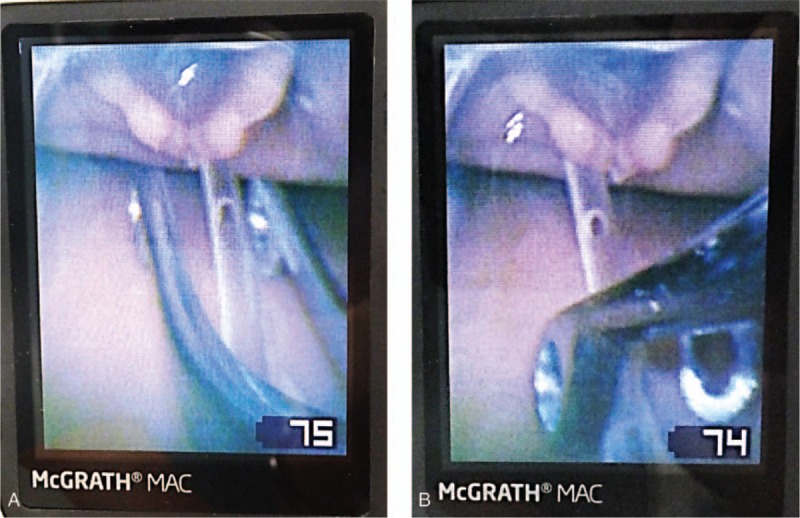
Depiction of NGT insertion using the SUZY curved forceps or the conventional Magill straight forceps under guidance of the McG videolaryngoscope. The tips of the SUZY forceps (A) can be more easily visualized on the McG monitor compared with the tips of the Magill forceps (B). The SUZY forceps can also be manipulated up to a deeper part of the pharynx and can advance the NGT for a longer distance compared with the Magill forceps. McG = McGRATH MAC, NGT = nasogastric tube.

The SUZY forceps are designed for the removal of pharyngolaryngeal foreign bodies,^[13,14]^ and their curved form fits the blade of McG. Therefore, the tips of the SUZY forceps can be clearly visualized on the McG monitor (Fig. 4). Our results also suggested that the SUZY forceps could advance the NGT for a longer distance per stroke (4.3 cm) compared with the Magill forceps (1.8 cm) during insertion from the pharynx to the esophagus. Thus, the SUZY forceps may be an effective tool for safe and relatively easy insertion of an NGT. Furthermore, longer distances per stroke and shorter NGT insertion times would lead to a decrease in the incidence of pharyngolaryngeal complications, because repeated forceps manipulations and a longer duration of contact with the mucosa can result in mucosal injury. Indeed, it has been reported that McG decreased the incidence of pharyngolaryngeal complications associated with the insertion of a transesophageal echocardiography probe.^[15]^

The use of videolaryngoscopes can improve the success rate for NGT insertion.^[7–11]^ However, an NGT cannot be directed to the esophagus using videolaryngoscopes alone. In addition, because videolaryngoscopes provide a narrower visual field compared with the Macintosh laryngoscope, clinicians often encounter difficulty in maintaining the forcep tips within the monitor. Therefore, NGT insertion under videolaryngoscopic guidance alone is not an ideal mainstream modality. The use of the SUZY forceps in conjunction with McG guidance can overcome these limitations according to our findings, which demonstrated that the SUZY forceps were easy to manipulate under McG guidance, even though none of the participants had used them before the study.

This study has some limitations. First, it was a manikin study and did not include actual clinical settings. Moreover, adverse events such as mucosal injury, edema, and bleeding could not be evaluated. Further study is needed to evaluate the efficacy and the safety of the SUZY forceps for NGT insertion. Second, while the SUZY forceps used in conjunction with McG proved effective for NGT insertion, the technique does not overcome the possibility of problems occurring at the esophagogastric junction. Because NGTs are usually designed to optimize patient safety, most of them are soft and occasionally do not bypass the lower esophageal sphincter. Various types of guidewires can be utilized to enable the NGT to bypass this junction, although this can result in trauma to the pharyngeal and esophageal mucosa.

In conclusion, the use of the SUZY forceps in conjunction with McG guidance facilitated NGT insertion with fewer manipulations and in a shorter time compared with the use of the Magill forceps in this manikin study. These results suggest that the use of the SUZY forceps under McG guidance is a secure and easy procedure for NGT insertion under direct visual guidance.

## Acknowledgments

The authors would like to thank Editage for providing editorial assistance.
